# The Forest behind the Tree: Phylogenetic Exploration of a Dominant *Mycobacterium tuberculosis* Strain Lineage from a High Tuberculosis Burden Country

**DOI:** 10.1371/journal.pone.0018256

**Published:** 2011-03-25

**Authors:** Maranibia Cardoso Oelemann, Harrison M. Gomes, Eve Willery, Lia Possuelo, Karla Valéria Batista Lima, Caroline Allix-Béguec, Camille Locht, Yves-Olivier L. Goguet de la Salmonière, Maria Cristina Gutierrez, Philip Suffys, Philip Supply

**Affiliations:** 1 Laboratory of Molecular Biology Applied to Mycobacteria, Oswaldo Cruz Institute, Fiocruz, Rio de Janeiro, Brazil; 2 INSERM U1019, Lille, France; 3 CNRS UMR 8204, Lille, France; 4 Univ Lille Nord de France, Lille, France; 5 Institut Pasteur de Lille, Center for Infection and Immunity of Lille, Lille, France; 6 Center of Scientific and Technological Development, Fundação Estadual de Produção e Pesquisa em Saúde, Porto Alegre, Brazil; 7 Institute Evandro Chagas, Belém, Brazil; 8 Department of Infection and Epidemiology, Institut Pasteur, Paris, France; The University of Hong Kong, Hong Kong

## Abstract

**Background:**

Genotyping of *Mycobacterium tuberculosis* isolates is a powerful tool for epidemiological control of tuberculosis (TB) and phylogenetic exploration of the pathogen. Standardized PCR-based typing, based on 15 to 24 mycobacterial interspersed repetitive unit-variable number of tandem repeat (MIRU-VNTR) loci combined with spoligotyping, has been shown to have adequate resolution power for tracing TB transmission and to be useful for predicting diverse strain lineages in European settings. Its informative value needs to be tested in high TB-burden countries, where the use of genotyping is often complicated by dominance of geographically specific, genetically homogeneous strain lineages.

**Methodology/Principal Findings:**

We tested this genotyping system for molecular epidemiological analysis of 369 *M. tuberculosis* isolates from 3 regions of Brazil, a high TB-burden country. Deligotyping, targeting 43 large sequence polymorphisms (LSPs), and the MIRU-VNTR*plus* identification database were used to assess phylogenetic predictions. High congruence between the different typing results consistently revealed the countrywide supremacy of the Latin-American-Mediterranean (LAM) lineage, comprised of three main branches. In addition to an already known RDRio branch, at least one other branch characterized by a phylogenetically informative LAM3 spoligo-signature seems to be globally distributed beyond Brazil. Nevertheless, by distinguishing 321 genotypes in this strain population, combined MIRU-VNTR typing and spoligotyping demonstrated the presence of multiple distinct clones. The use of 15 to 24 loci discriminated 21 to 25% more strains within the LAM lineage, compared to a restricted lineage-specific locus set suggested to be used after SNP analysis. Noteworthy, 23 of the 28 molecular clusters identified were exclusively composed of patient isolates from a same region, consistent with expected patterns of mostly local TB transmission.

**Conclusions/Significance:**

Standard MIRU-VNTR typing combined with spoligotyping can reveal epidemiologically meaningful clonal diversity behind a dominant *M. tuberculosis* strain lineage in a high TB-burden country and is useful to explore international phylogenetical ramifications.

## Introduction

Despite the existence of effective antituberculosis drugs for the last 60 years, tuberculosis (TB) continues to be a major threat worldwide. Eighty percent of the estimated 9.4 million new TB cases arising each year occur amongst the 22 countries with the highest global TB burden [1]. With 87,000 new TB cases reported in 2009, Brazil ranks 19^th^ among these countries. In Latin America, Brazil and Peru together account for approximately 45% of all TB cases [1]. The TB threat in Brazil is further strengthened by the relatively high frequency of HIV co-infection, as, according to the latest WHO report [1], about 14% of the 72% TB patients tested for HIV infection were found to be HIV-positive, and an estimated 20% of people living with HIV-AIDS have pulmonary TB [2].

Molecular typing of *Mycobacterium tuberculosis* is a powerful adjunct to TB control, e.g. to monitor the disease transmission, to detect or confirm outbreaks and laboratory error/cross-contamination, to distinguish endogenous reactivation from exogenous reinfection and to identify the clonal spread of successful clones, including multi-drug-resistant ones [3]. Molecular typing can also help distinguishing between members of the *M. tuberculosis* complex, which might have vital clinical implications under certain circumstances [4]. However, its application in high TB-burden countries has been hampered hitherto by constrained resources, technical limitations of standard IS*6110* fingerprinting [5], and, in general, by the dominance of geographically specific, genetically homogeneous strain lineages (e.g. [6]), which renders molecular discrimination of unrelated clones difficult.

Among PCR-based approaches developed for *M. tuberculosis* genotyping, MIRU-VNTR typing, optionally combined with spoligotyping, has been shown a valuable routine alternative to IS*6110* RFLP. Initially based on 5- [7] or 12-locus formats [8], [9], this method allows high-throughput analysis of clinical isolates [10] and is now widely carried out, including in the universal genotyping system in the USA [11]. Recently, an optimized 15 to 24-locus MIRU-VNTR typing scheme has been proposed for international standardization [12]. Several reports have shown its appropriateness for population-based studies of TB transmission in high-income countries [13], [14], [15], [16]. However, the applicability of this standardized system has so far not been tested in high TB-burden countries, except in a geographically restricted South African setting with extreme epidemiological conditions [17] or in certain Chinese regions with a specific *M. tuberculosis* Beijing strain population [18]. More specifically, only a few, limited molecular epidemiological studies have been performed in Brazil [19], [20], [21], [22], [23], [24].

In addition to their use for tracing TB transmission at the strain level, MIRU-VNTR markers are also phylogenetically informative, especially in the 24-locus format, and can therefore be used to predict groupings into strain lineages [12], [25]. Strain lineage information is useful from an epidemiological control perspective, as it can provide indications on the source of TB cases (ongoing transmission versus reactivation or importation of an infection acquired abroad) [26]. Such information is valuable as well for the development of new tools for TB control [27]. However, a recent report argued against the use of MIRU-VNTR typing and spoligotyping for phylogenetic identification [28].

Here, we tested the applicability of this standardized MIRU-VNTR system, combined with spoligotyping for molecular epidemiological analyses of 369 *M. tuberculosis* isolates from three regions of Brazil. Deligotyping, a convenient method based on PCR interrogation of pre-selected large sequence polymorphisms (LSPs) [29] comprising reference phylogenetic markers [30], [31], and the recent MIRU-VNTRplus identification database [32] were used in addition to assess strain lineage predictions, based on MIRU-VNTR and spoligotyping. Finally, the SpolDB4 database was used to investigate international spread of clonal branches predicted on the basis of phylogenetically consistent spoligotype signatures as defined by monophyletic MIRU-VNTR groupings.

## Materials and Methods

### Ethics statement

Surveys in the study regions were approved by the Institutional Review Boards of the Federal University of Rio de Janeiro and Weill Medical College of Cornell University of New York, and the Ethics Committees for Research of Fundação Estadual de Produção e Pesquisa em Saúde (Porto Alegre) and Institute Evandro Chagas (Belém).

### Patient isolates, and genomic DNA

A convenience sampling of 399 *M. tuberculosis* clinical isolates was initially used for the present study. *M. tuberculosis* isolates were obtained from clinically confirmed TB patients, presenting pulmonary or extrapulmonary forms, diagnosed from 2000 to 2003 and reported to community health centers located in Rio de Janeiro, Porto Alegre, and Belém, the three Brazilian cities included in the study. The collected samples were treated at the TB referral hospitals of the respective cities. As drug-susceptibility testing (DST) is not systematically performed in Brazil, detailed DST data were not available, nor were patient epidemiological and clinical data. The isolates were identified as *M. tuberculosis* according to standard phenotypic criteria. PCR analyses were performed using frozen aliquots of thermolysates, as described previously [2], or genomic DNA extracted using CTAB [33]. Thirty isolates were excluded for various reasons, including insufficient DNA quality, probable clerical errors, sample contamination and/or mixed infection, as detected by double alleles in two or more MIRU-VNTR loci [34], [35]. A final sample of 369 isolates, comprising 177 isolates from Rio de Janeiro, 120 from Porto Alegre and 72 from Belém was thus retained for the analysis. These isolates originated from different patients, except 8 that each corresponded to a second isolate obtained from one of 8 different patients from Rio de Janeiro. Genomic DNA obtained from *M. tuberculosis* H37Rv and/or *M. bovis* BCG Pasteur reference strains was used as controls in all experiments.

### Molecular methods

Twenty-four-locus-based MIRU-VNTR typing was applied using a 96-capillary-based ABI 3730 genetic analyzer as described in [34] and [12]. Calibration and quality control procedures followed were shown to result in 100% MIRU-VNTR typing result reproducibility at intra- and interlaboratory levels by a recent international external quality control organized by the RIVM/ECDC (De Beer et al., in preparation). Spoligotyping was used, as previously described by Kamerbeek et al. [36]. Spoligotyping results of the isolates from Rio de Janeiro were from the study of Lazzarini et al. [2]. MIRU-VNTR, spoligotyping and deligotyping were each performed blindly from the results obtained using the two other methods and from the serial isolate information.

### Verification of LSPs in clinical isolates

Deligotyping was performed as described by Goguet de la Salmonière *et al*. [29]. Oligonucleotides, the corresponding genomic regions, genes and their Rv numbers are listed in Table 1.

**Table 1:** Deligotyping genomic deletion targets and reference large sequence polymorphisms.

Blotter Lane	Target gene or genomic region in H37Rv (alias gene name)	Reference LSP^a^	Lineage specificity^a^
1	*Rv0921*		
2	*Rv2812*		
3	*Rv3446c (mtrA)*		
4	*Rv2739c*		
5	*Rv0195*		
6	*Rv0405 (pks6)*		
7	*Rv0571c*		
8	*Rv3901c*		
9	Rv1189 (*sigI*)	RD 142	East-Asian
10	*Rv1355c (moeY)*		
11	*Rv1512 (epiA)*		
12	*Rv1582c*		
13	*Rv1672c*	RD 150	East-Asian
14	*Rv1755c (plcD)*		
15	*Rv1761c*		
16	*Rv1878 (glnA3)*		
17	*Rv1907c*		
18	*Rv1927*		
19	*Rv1976c*		
20	*RV1985c*		
21	*Intergenic*		
22	*Rv1994c (cmtR)*	RD 174	Euro-American
23	*Rv2002 (fabG3)*		
24	*Rv2124c (metH)*		
25	*RV2275*	RD 182	Euro-American
26	*RV2314c*	RD 183	Euro-American
27	*Rv2349c (plcC)*		
28	*RV2351c (plcA/mtp40antigen)*		
29	*Rv2406c*	RD 193	Euro-American
30	*valU (tRNA-Val)*		
31	*Rv3084 (lip R*)	RD 219	Euro-American
32	*Rv3121 (cyp141)*		
33	*Rv3402c*		
34	*Rv3425 (PPE57)*		
35	*Rv3429 (PPE59)*		
36	*Rv3519*		
37	*RV1769*		
38	*Intergenic*		
39	*Rv3617 (ephA)*		
40	*Rv3651*	RD 239	Indo-Oceanic
41	*Intergenic*		
42	*RV3766*		
43	*Rv3875 (esat-6/esxA)*		

a According to Gagneux et al. [30].

### Computer-assisted analysis of the patterns

Spoligotyping, deligotyping and MIRU-VNTR profiles were analyzed and used to generate dendrograms, using the Bionumerics package version 4.5 (Applied Maths, St-Martin-Latem, Belgium). Dendrograms based on MIRU-VNTR patterns were generated using the categorical coefficient and neighbor joining method and were rooted using a “M. prototuberculosis*”* C/D genotype (alias *M. canetti*) as outgroup [37]. A strain-cluster was defined as two or more patients infected by isolates having identical genotypes, depending on the method(s) considered. For cluster analysis, in cases of isolates with clonal variants, the single locus displaying a double allele was not considered for genotype comparisons [35].

### Use of MIRU-VNTR*plu*s database

Spoligotyping and MIRU-VNTR patterns were submitted to MIRU-VNTRplus (www.miru-vntrplus.org) [38] for lineage identification, using the strategy described in [32], [38]. Briefly, best-match analysis was performed based on MIRU-VNTR typing combined with spoligotyping, using a distance cut-off of 0.17, followed by tree-based analysis based on 24 MIRU-VNTR loci to maximize and further support predictions.

## Results

### Study population

Rio de Janeiro, Belém and Porto Alegre are the capitals of Rio de Janeiro, Pará and Rio Grande do Sul states and are located in the North, South East and extreme South of Brazil, respectively. According to the federal health department (http://portal.saude.gov.br/portal/arquivos/pdf/tuberculose.pdf), Rio de Janeiro, Pará and Rio Grande do Sul ranked second, eighth and fifth in TB incidence in 2002 among the 26 Brazilian states, cumulating together approximately 21,000 confirmed TB cases. A conveniently selected sample of 361 *M. tuberculosis* isolates, all from different patients from the three study cities, was finally retained for baseline analysis of genotypic diversity in these cities. Eight serial isolates, each corresponding to a second isolate obtained from one of 8 different patients from Rio de Janeiro, were also included in order to explore the clonality of *M. tuberculosis* infection in the studied population and to test the consistence of the genotyping results.

### MIRU-VNTR-spoligotype-based cluster analysis

The 24-locus MIRU-VNTR genotypes and spoligotypes were fully conserved between the two isolates within each of the eight pairs of serial isolates tested.

**Figure 1:**
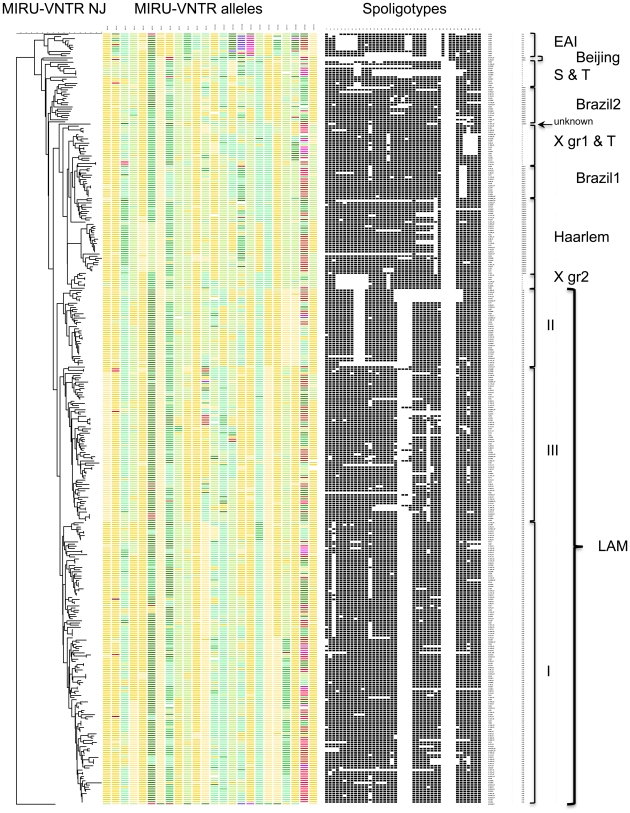
Genotypic diversity of *M. tuberculosis* isolates from 3 Brazilian regions. Colour-coded 24-locus MIRU-VNTR alleles and spoligotypes from 361 isolates are represented. A MIRU-VNTR-based dendrogram was generated using the neighbor-joining algorithm and rooted using a *M. prototuberculosis* C/D genotype (alias *M. canettii*) as outgroup. *M. tuberculosis* strain lineages and branches shown at the right were identified by analyzing the congruence of MIRU-VNTR typing and spoligotyping results within this collection and submitting the isolate genotypes to the MIRU-VNTR*Plus* identification database (see text). X gr1 and gr2 (groups 1 and 2) correspond to X del26/RD183 and Xdel29/RD193 in Fig. 3, respectively.

In contrast, the set of 361 isolates from different patients displayed highly diverse MIRU-VNTR genotypes (Table 2 and Fig. 1). Three-hundred and seven distinct genotypes were detected using the full 24 MIRU-VNTR locus set, including 37 cluster patterns and 270 unique patterns. Consistent with previous results obtained in Europe [12], [13], [15], [16], only a minority of these MIRU-VNTR clusters was split by the addition of secondary spoligotyping, resulting in a total of 321 distinct patterns, reducing the number of clusters to 28 and increasing the number of unique isolates to 293. Remarkably, the use in combination with spoligotyping of the discriminatory subset of 15 MIRU-VNTR loci generated only a handful less genotypes (315) than the full set of 24 loci combined with spoligotyping, again in line with previous findings [12], [13], [16].

**Table 2:** Discriminatory powers of different genotyping methods used.

Method	Total number of types	Unique types	Clustered types
MIRU-VNTR 24 + spoligotyping	321	293	28
MIRU-VNTR 15 + spoligotyping	315	286	29
MIRU 24 alone	307	270	37
MIRU 15 alone	295	256	39
MIRU 12 + spoligotyping	268	220	48
MIRU 12 alone	185	123	62
Spoligotyping alone	145	104	41

In contrast, only 268, 185 and 145 patterns were observed with the initial set of 12 MIRU-VNTR loci combined with spoligotyping, the 12 MIRU-VNTR locus set alone or spoligotyping alone, respectively.

**Figure 2:**
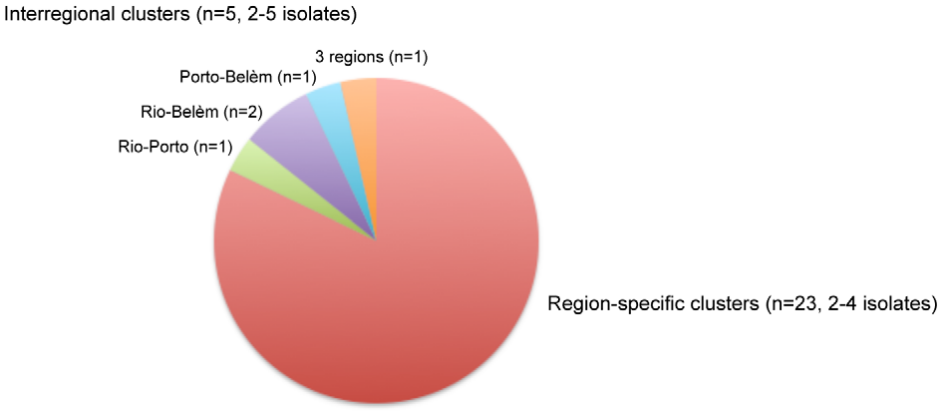
*M. tuberculosis* strain cluster distribution among three Brazilian regions. Molecular clusters are defined by isolates sharing identical 24-locus MIRU-VNTR genotypes and spoligotypes.

The largest cluster defined by 24-MIRU-VNTR typing combined with spoligotyping included 5 isolates, while 20 of the remaining 28 clusters were composed of only two isolates. Noteworthy, 23 clusters were exclusively composed of patient isolates from a same region, while only five of them, including the largest one, comprised isolates from several different regions (Fig. 2).

### Strain lineage identification based on MIRU-VNTR and spoligotyping

Because of the highly clonal nature of *M. tuberculosis* [31], [39], [40], the analysis of the congruence between sets of fully independent markers is a relevant approach to identify strain lineages (e.g. [41], [42]). Strain lineage distribution was therefore determined in the collection (Table 3 and Fig. 1), firstly by constructing a neighbour-joining tree based solely on 24-MIRU-VNTR typing results and then by examining its congruence with the corresponding spoligotyping results. The results were subsequently tested by submitting the isolate genotypes to the MIRU-VNTRplus identification database. This database permits lineage prediction of submitted isolates, by comparing their MIRU-VNTR and spoligotyping profiles to those of reference strains, whose lineages are also defined by reference LSPs and SNPs.

**Table 3:** Distribution of *M. tuberculosis* lineages in the study.

	All isolates, n = 361 (%)	Deligotyping selection, n = 137 (%)
Lineage or branch^a^		
Indo-Oceanic/East-African Indian	11 (3)	10 (7.3)
East Asian/Beijing	2 (0.5)	2 (1.5)
Euro-American/S	9 (2.5)	4 (2.9)
Euro-American/T	7 (2)	3 (2.2)
Euro-American/X	19 (5.2)	10 (7.3)
Euro-American/Brazil-1	16 (4.4)	7 (5.1)
Euro-American/Brazil-2	20 (5.5)	2 (1.5)
Euro-American/Haarlem	35 (9.7)	12 (8.7)
Euro-American/LAM	241 (66.2)	86 (62.8)
Unknown	1 (0.2)	1 (1.4)

a Nomenclatures corresponding to Gagneux et al. [30]/Brudey et al. [48], except for Brazil-1 and -2 named according to this study.

Remarkably, this analysis disclosed that 348 (96.4%) of the 361 isolates belong to the Euro-American super-lineage [27], which was itself strongly predominated by the LAM lineage represented by 241 (66.2% of the total) isolates. Within the LAM lineage, three large subgroups were clearly predicted and accordingly named LAMI to LAMIII.

Other, minor members of the Euro-American super-lineage included isolates with S, X and T spoligotypes, where the non-members of the Euro-American super-lineage were only comprised of 11 East-African Indian and only two Beijing (alias East-Asian) isolates. Among the minor Euro-American members, MIRU-VNTR-based groupings predicted the existence of two separate branches of X-spoligotype strains, named X group 1 and 2. Two groups with T spoligotypes were respectively named Brazil 1 and 2, based on their specific MIRU-VNTR groupings.

One hundred and thirty-seven isolates, representing the diversity of the different lineages and subgroups identified on the basis of MIRU-VNTR typing and spoligotyping (Table 3), were selected for deligotyping analysis.

### LSP interrogation and congruence with MIRU-VNTR and spoligotyping

Deligotyping interrogates 43 genomic regions prone to LSPs among *M. tuberculosis* complex strains [29]. The targeted markers include 8 LSPs that were previously used as gold standards to define different branches of the Euro-American super-lineage (deligotyping probes 22, 25, 26, 29, and 31), two specific branches of the East Asian lineage (probes 9 and 13) and the Indo-Oceanic lineage (alias East-African-Indian in spoligotyping nomenclature; probe 40) [30], [43]. These LSPs are referred to as reference LSPs hereafter.

**Figure 3:**
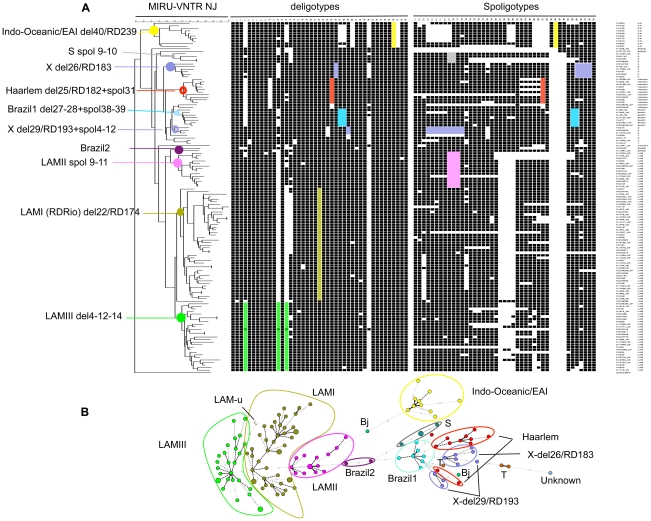
Congruence analysis between MIRU-VNTR typing, deligotyping and spoligotyping. A selection of 137 isolates was used, representing the diversity of the different lineages and subgroups predicted based on MIRU-VNTR typing, spoligotyping and the MIRU-VNTR*Plus* database (see text, Table 3 and Fig. 1). A. A MIRU-VNTR-based dendrogram was generated using the neighbor-joining algorithm and rooted using a *M. prototuberculosis* C/D genotype (alias *M. canettii*) as outgroup. Solid coloured circles on tree nodes indicate MIRU-VNTR groupings that are monophyletic when compared to LSP-based (deligotyping) and/or (when deligotyping was not informative) spoligotyping-based groupings. Partially monophyletic groupings are indicated by coloured rings. B. MIRU-VNTR-based minimum spanning tree. The same isolates were used as in the neighbor-joining tree. Colours and grouping names correspond to those of panel A. Distances between circles are proportional to the number of allele differences between MIRU-VNTR genotypes; circle sizes are proportional to the numbers of isolates sharing an identical genotype. Del, deligotyping probe; RD, region of difference (reference LSP, see Table 1); spol, spoligotyping spacer; LAMu, unclassified LAM isolate. Color codes of phylogenetic groups (see text for further description): yellow, Indo-Oceanic (LSP)/EAI (spoligotyping); grey, S; light purple, X; red, Haarlem; blue, Brazil 1; dark purple; Brazil 2; pink, LAM II; khaki, LAM I; green, LAM III.

Among the selected 137 *M. tuberculosis* isolates from Brazil, deligotyping indicated LSPs in a total of 28 of these regions (Fig. 3). Among them, seven were detected in a single isolate, while 21 were found in two or more isolates.

Out of the 8 reference phylogenetically informative LSPs, the two East Asian-specific ones were found to be non-informative in this isolate population, indicating that the two Beijing strains identified are not part of RD142 or RD150 branches [30]. These two isolates were not grouped together in this MIRU-VNTR-based tree nor in the minimum spanning tree, although they were in the first tree including the total collection (Fig. 1), linked to a distant relationship rendering their linkage more vulnerable to the composition of the strain sample used. Surprisingly, a deletion was indicated for East Asian-specific probe 9 for a single isolate, although convergently classified as LAM by MIRU-VNTR typing and spoligotyping. Results obtained with the probes matching the six other reference LSPs were fully concordant with the lineage predictions based on MIRU-VNTR typing complemented by spoligotyping. All the isolates monophyletically grouped by MIRU-VNTR typing into the East-African Indian lineage (spoligotyping nomenclature) specifically displayed negative signals with probe 40, matching Indo-Oceanic-specific RD 239 LSP. Likewise, deligotypes matching either of the 5 targeted Euro-American-specific LSPs were specifically observed among isolates predicted to belong to different branches of this super-lineage.

High congruence between MIRU-VNTR- and reference LSP-based groupings was also generally seen at higher resolution (Fig. 3). In the Euro-American super-lineage, the 44 isolates displaying negative signals against probe 22, matching reference RD 174 LSP, were all monophyletically grouped by MIRU-VNTR typing into one major branch of the LAM lineage, designated as LAM-I. Similarly, the six strains with probe 26-negative deligotypes matching reference LSP RD 183 (and specifically absent spoligotype spacers 39 to 42) were monophyletically grouped as a X-spoligotype subgroup by the MIRU-VNTR results (consistently corresponding to X group 1 in Fig. 1). Groups of isolates with probe 25- or probe 29-negative deligotypes matching two other Euro-American reference LSPs were not completely monophyletic by MIRU-VNTR typing, but 10 out of 12, and 3 out of 5 isolates, respectively, were part of one same specific group (respectively classified as Haarlem, and X group 2 or T subgroup in Fig. 1). In each of these two cases, the few remaining isolates were part of a proximal group in this strain set but had nevertheless the same Haarlem or X classification, based on previous MIRU-VNTR*Plus* best-match and/or tree-based analysis (see above).

Such high congruence was also generally seen when additional deligotype probes beyond those matching the 8 reference LSPs were included in the analysis. Some of these probes were not specific of a (sub)lineage, but were nevertheless informative when taken in combination. Moreover, some spoligotype signatures could also be used as an independent reference for congruence analysis, especially when LSP analysis was not informative. For instance, all the isolates grouped into the EAI lineage by MIRU-VNTR typing and reference probe 40 were also specifically matched by probe 41-negative deligotypes, and systematically as well by probes 4 and 30 (albeit these were not specific to EAI). Likewise, the six X isolates monophyletically grouped by MIRU-VNTR typing and reference probe 26 were concordantly matched by probes 12 and 34. An additional monophyletic set, named Brazil 1, of the same broad group was supported by systematic matches with probes 27 and 28, in addition to deletions of spoligotype spacers 38–39. In the dominant LAM lineage, in addition to the monophyletic branch jointly defined by MIRU-VNTR typing and reference probe 22, two other main branches identified by MIRU-VNTR typing were clearly supported by deligotyping or spoligotyping results, and were accordingly called LAM II and III. LAM II formed a monophyletic group independently matched by the specific deletion of spoligotype spacers 9 to 11. LAM III was matched by the specific combination of deligotype probes 4, 12 and 14 (with a single apparent outlier), or by default as LAM genotypes nor sharing reference LSP 22, nor the deletion of spoligotype spacers 9 to 11.

## Discussion

In order to explore the informative value of 24-locus MIRU-VNTR typing supplemented by spoligotyping in a high TB-burden country, we analyzed a baseline sample of 369 *M. tuberculosis* isolates from three Brazilian regions. Predictions of phylogenetic classifications based on these markers were assessed by using LSP phylogenetical markers, as interrogated by deligotyping.

**Figure 4:**
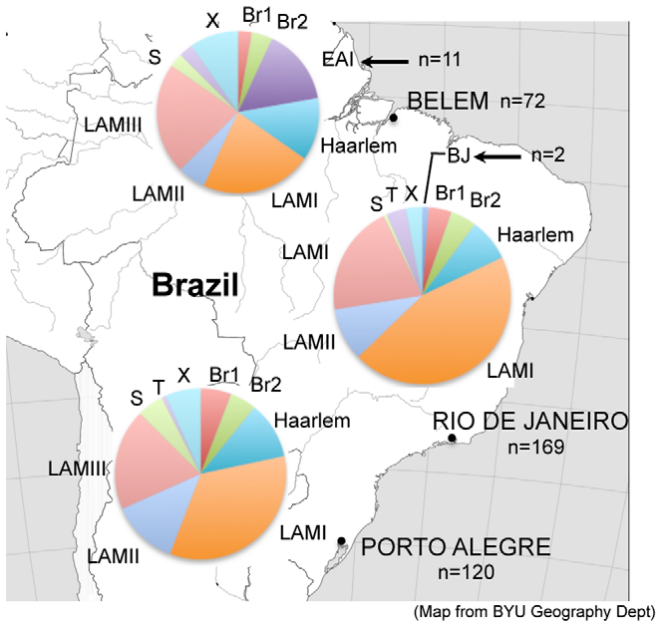
*M. tuberculosis strain* lineage distribution among three Brazilian regions. Lineage distributions result from the congruence analyses shown in Fig. 1 and 3. One isolate from Rio De Janeiro was of an unknown lineage/branch. Arrows indicate region-specific lineages. Br1 and Br2, Brazil 1 and 2, respectively.

This analysis indicates extreme dominance in Brazil of the *M. tuberculosis* Euro-American super-lineage, itself strongly predominated by the LAM lineage, representing 96.4% and 66.8%, respectively, of all isolates. Although this strain collection is not necessarily representative of all strains circulating in the country, our results suggest that, similar to other high TB-burden countries (e.g. [6], [44], [45]), Brazil has a highly restricted array of strain lineages, in contrast to European or North American regions (e.g. [13], [16], [46]). This contrast in diversity reflects the association between *M. tuberculosis* strains and their human host populations and demographic history [25], [30], [31], [47], and can be explained by a concentration of TB cases among often recently immigrated, foreign-born patients of cosmopolitan origins in Europe and North America, as opposed to more ubiquitous distribution of cases in the general, more previously settled population in high TB-burden countries. In the case of Brazil, the LAM lineage supremacy likely mirrors the persisting influence of the prolonged, major immigration from Southern Europe and Africa, where this lineage is widespread as well [13], [48]. However, we noticed the significant presence of East-African Indian/Indo-Oceanic (according to the spoligotype or LSP nomenclature, respectively) lineage, exclusively among the strains from Belém, Para (11/72, Fig. 4), indicating a geographically stippled influence from other regions of the world. In contrast, W/Beijing isolates were barely represented (2/361, both in Rio) in this Brazilian collection, in agreement with previous studies from South America (for review, see [49]).

The LAM spoligotype “clade” is one of the 6 major *M. tuberculosis* spoligotype families and a major member of the Euro-American super-lineage, particularly present in South America [27], [28], [48]. In the spolDB4 world spoligotype database, the LAM-classified spoligotypes even outnumber the W/Beijing spoligotypes [48]. Paradoxically though, the genetic consistency and global composition of the LAM spoligotype family is much less studied than that of the W/Beijing family [2], [50]. Especially in regions where this lineage prevails, a better definition of these parameters is needed both for elucidating the evolutionary history of *M. tuberculosis*, and as a basis for efficient molecular-guided TB control and surveillance. Our analysis of congruence between the MIRU-VNTR, spoligotype and LSP results demonstrates that the LAM lineage is composed of at least three different main clonal branches, referred to as LAM I to III in this study.

The LAM I branch is characterized by deligotype probe 22, matching RD174. This deletion has been recently shown to co-segregate with another specific genomic deletion called RDRio in other LAM strains, referred to as the RDRio branch. RDRio strains were initially identified as being prevalent in Rio de Janeiro, and subsequently found to be present in other regions of the world, even outside the Americas [2], [50]. The two other LAM branches were not matched by individual specific LSPs in this analysis, but by monophyletic congruence of MIRU-VNTR grouping and the specific combination of deligotype probes 4, 12 and 14 (LAM III), or by a specific spoligotype signature (absence of spacers 9 to 11, in addition to classical LAM spoligotype signature(s)) (LAM II). The latter signature is designated as LAM 3 spoligotype subclade, represented by >400 isolates obtained from the American, European, African and even the Oceanic continents in SpolDB4 [48]. Within the limits of this study, the monophyletic grouping by MIRU-VNTR typing of the isolates with the latter signature supports the phylogenetic consistency of this spoligotype subclade designation. Taken together, these results thus indicate that, similar to the LAM I/RDRio branch, the LAM II branch has a large geographic distribution, well beyond Brazil and South America. In contrast, the LAM III branch had no such common specific spoligotype signature that could help exploring its worldwide distribution based on SpolDB4, as several spoligotypes even intriguingly displayed one or more of the spacers 21 to 24 that normally define the general LAM spoligo-prototype [48]. Although samples were unavailable for repeating spoligotyping (as for another outlier by deligotyping, see below), the fact that the latter exceptions are branch-specific strongly suggests that these atypical spoligotype patterns are not due to a technical problem. A similar deviation from the general LAM spoligo-prototype was also recently indicated by SNP analysis (see strain LA19 in Fig. 2 of ref. [28]).

This LAM-lineage supremacy conceals a marked multiplicity of distinct clones in the studied strain population. As many as 321 distinct genotypes based on MIRU-VNTR and spoligotypes were identified among the 361 isolates from different patients, and the 28 clusters found were mostly (n = 20) composed of only two isolates. No attempt was made to compare these genotypes and clustering with those that could have been obtained by using standard IS*6110* RFLP, due to the technical difficulties associated with the latter technique. However, the discriminatory power observed in this large strain sample by using MIRU-VNTR and spoligotyping is already so high that a significant increase in the number of genotypes as defined by IS*6110* RFLP would not be expected. Likewise, due to difficulties inherent to high TB incidence settings (potential multiple infection sources, resource prioritization, difficulty of defining full population-based collections, etc.), no epidemiological or contact tracing data was available to independently and systematically evaluate the epidemiological significance of the strain clusters identified. Nevertheless, the observation that 23 of the 28 clusters identified were exclusively composed of patient isolates from a same city constitutes a substantial surrogate support for such epidemiological significance. Indeed, such cluster composition is consistent with expected patterns of mostly local (e.g. intra-city or -region) TB transmission, as inferred from other large multi-site studies where clustered isolates are generally restricted to a single geographic site [51]. Furthermore, the finding that the 24-locus MIRU-VNTR genotypes and spoligotypes were fully conserved between the two isolates of each of the eight pairs of serial isolates from a same patient tested is consistent with the well-documented clonal stability of these markers during chronic infection and in transmission chains [12], [52], [53], [54], and an expected more frequent scenario of monoclonal infection over different disease episodes or sputum samples compared to settings with higher TB incidence (e.g. [55]). Meticulous calibration studies found that standard MIRU-VNTR genotypes changed in about less than 5% of >200 isolates from population-based clusters with proven epidemiological links and sets of serial isolates (covering time periods of up to seven years), and never by more than a single locus [12], [52], [53], [54]. These data, combined with refined analyses of single-locus variation frequencies in a population-based dataset of >2700 isolates, lead to an estimate of mean mutation rate per year and per locus of 10^−4^ [25]. Altogether, these results thus indicate that the small sizes of the strain clusters observed here (at most 5 isolates) do likely not result from high mutation rates (i.e. high instability) of the combined markers relative to the transmission rate of a given clone, but reflect well in most instances the numerous truly epidemiologically-unrelated, clonally distinct isolates existing in the study sample.

In addition to their primary use for molecular discrimination and tracing at the strain level, molecular epidemiological markers can also serve to predict genetic lineages, which is useful both for TB control and research. A recent report [28] argued against the use of MIRU-VNTR typing or spoligotyping for such purposes, based on comparisons of detailed phylogenies obtained with each of the two marker sets separately with those based on LSPs and SNPs. However, rather than inferring exact phylogenies for each isolate, a more generic classification into main *M. tuberculosis* complex lineages is most often looked for as a secondary information by most users of molecular epidemiological markers, e.g. as a surrogate information for identifying sources of TB infection (local vs imported from abroad) (e.g. [26]) or risks of drug resistance in certain geographic regions [56]. In this study, the combined use of 24-locus MIRU-VNTR typing and spoligotyping for best-match analysis in MIRU-VNTRPlus, followed by tree-based analysis solely using 24-locus MIRU-VNTR typing as described in [32] resulted in predictions of the main lineages represented (Euro-American, Indo-Oceanic) that were almost completely verified by secondary LSP interrogation (except a single potential outlier with an unexpected East Asian deligotype pattern). Furthermore, the MIRU-VNTR tree-based analysis was highly congruent with deligotyping and/or spoligotyping results for defining branches within the Euro-American superlineage and its LAM component. Most MIRU-VNTR groups were monophyletic when compared to informative LSPs (i.e. lineage-specific and present in >1 isolate) and/or spoligotype signatures. LSP-based results also confirmed the existence of two distinct X spoligotype groups predicted by MIRU-VNTR groupings (see Fig. 1 and 3). Only a few outliers were observed, for which technical problems linked to spoligotype or deligotype hybridization could not be excluded. In agreement with previous studies [12], [13], [25], [32], these results thus show that 24-locus MIRU-VNTR typing is of high predictive value, albeit not fully deterministic [28], for classifying isolates into lineages. Recently developed user-friendly Bayesian network tools especially adapted to analyse allelic distributions of MIRU-VNTR markers may even further increase the reliability of MIRU-VNTR-based classifications [57].

Not surprisingly, deligotyping had a much lower resolution power compared to 24-locus- based MIRU-VNTR typing combined with spoligotyping (43 versus 134 profiles, respectively, among the 137 isolates tested with both methods; see Fig. 3). This finding is consistent with the phylogenetic nature of the LSP markers interrogated by the former method, implying slow mutation rates restricting their use to identification at lineage level [31] in contrast to MIRU-VNTR typing offering discrimination at strain level.

Because the discriminatory powers of individual MIRU-VNTR markers vary according to the strain lineage [12], [28], Comas et al. [28] recommended to use only small lineage-specific subsets (i.e. 3 to 9) of the most discriminatory markers, after pre-identification of the lineage based on SNPs. We tested the potential impact of applying such a strategy on the resolution power of MIRU-VNTR typing on the 241 LAM isolates of this study, alternatively identified by Euro-American-specific LSPs and MIRU-VNTRPlus analysis. Using the subset of 9 loci specifically recommended by Comas et al. for the Euro-American super-lineage (comprising the LAM lineage) resulted in only 151 types, as compared to 192 and 202 types obtained with 15 and 24 loci. This result indicates that this dual, restrictive SNP/VNTR strategy cannot efficiently substitute for the full MIRU-VNTR set for discrimination at the strain level. This observation can easily be explained by the fact that only 97 strains were studied for the entire MTBC by Comas et al., including thus a few strains for most lineages (e.g. 5 representatives for the whole LAM lineage). Given the intrinsic stochastic component of VNTR changes and the likely numerous ramifications within each lineage, such a restricted representation could not embrace the MIRU-VNTR allelic ranges existing in each lineage, even with selected genetically distant representatives. Moreover, the above strategy did not account for the redundancy existing among the most discriminatory loci and the significant collective contribution of other, less variable but non-redundant loci. In contrast, the 15 to 24 MIRU-VNTR loci retained for standardization were selected to buffer against lineage- and branch-specific variations observed on a collection of >800 strains from >50 countries, including numerous representatives of the known principal lineages of the *M. tuberculosis* complex (e.g. 71 LAM isolates). Furthermore, this selection was based on analysis of single-locus variations, to account for redundancy [12].

In conclusion, this study suggests that the use of 24-locus MIRU-VNTR typing complemented by spoligotyping may be an effective strategy for molecular epidemiological applications in Brazil and probably in the many other regions of the world where LAM strains are common. These results thus tend to extend conclusions from population-based studies in low incidence settings with different, more assorted strain populations [13], [15], [16]. Interestingly, similar observations have also recently been obtained in a strain population from another region subjugated by another single *M. tuberculosis* lineage, i.e. Korea, by the Beijing lineage [58]. Therefore, to which extent a few additional, but less robust hypervariable MIRU-VNTR loci would need to be applied in a second-line investigation for refined discrimination in some specific cases remains an open issue ([12] and e.g. [17], [18], [59], [60], [61], [62]). Furthermore, although loci with the highest evolutionary rates can be prioritized within lineages [12], a full MIRU-VNTR locus set cannot be efficiently replaced to discriminate at the strain level by a dual strategy based on SNPs and only small, lineage-specific sets of MIRU-VNTR markers, as previously proposed [28]. Finally, this study confirms the highly predictive albeit not fully deterministic value of 24-locus MIRU-VNTR data for high-resolution, exploratory classification of isolates into MTBC strain lineages and sublineages [12], [28], [32]. These findings therefore suggest the following strategy to efficiently meet the objectives of most molecular epidemiological users: once discriminated at the strain level by full or prioritized MIRU-VNTR typing, classification of an isolate can be confirmed if necessary by the use of a few LSP or SNP markers [28], selected based on MIRU-VNTR lineage predictions.
